# Effect of long-term heat stress on structure and function of epidermal tissues in needles of treeline conifer seedlings

**DOI:** 10.1093/treephys/tpag080

**Published:** 2026-06-11

**Authors:** Giuseppe Tiloca, Othmar Buchner, Notburga Gierlinger, Gilbert Neuner

**Affiliations:** Environmental Sensing and Modelling Unit, Luxembourg Institute of Science & Technology LIST, Avenue des Hauts-Forneaux 5, Esch-sur-Alzette 4362, Luxembourg; Department of Natural Sciences and Sustainable Resources, Institute of Biophysics, Boku University, Muthgasse 11, Vienna 1190, Austria; Unit of Functional Plant Biology, Department of Botany, University of Innsbruck, Sternwartestr. 15, 6020 Innsbruck, Austria; Department of Natural Sciences and Sustainable Resources, Institute of Biophysics, Boku University, Muthgasse 11, Vienna 1190, Austria; Unit of Functional Plant Biology, Department of Botany, University of Innsbruck, Sternwartestr. 15, 6020 Innsbruck, Austria

**Keywords:** cell wall, cuticle, flavonoids, leaf minimum conductance, lignin

## Abstract

Tree seedlings at the treeline face various environmental extremes; in particular, their needles can heat up close to thermal limits. Unlike mature-tree needles, treeline conifer seedlings resist heat-induced water loss better, even though their cuticle lacks an outer layer. To determine whether these differences are due to ontogenetic factors or acclimative responses to higher heat loads, conifer seedlings were exposed to a controlled in situ long-term heat treatment. By comparing the rate of water loss through the cuticle (minimum diffusive conductance, *g*_min_), cuticle thickness and chemical micro-composition of control versus heated seedlings of deciduous *Larix decidua* Mill. and evergreen *Picea abies* (L.), we intended to assess the acclimative plasticity of these traits. We also aimed to identify specific heat-induced changes in cuticle structure and function. The controlled, in situ, long-term heat treatment lasted for 6 weeks with air temperature set 10–15 K above the ambient air (controls) with a set maximum leaf temperature of 42 °C. This heat treatment significantly reduced *g*_min_ in *L. decidua* but it was not significant in *P. abies*. In both species, exposure to heat induced an increase in cuticle thickness, as well as provoking species-specific microchemical responses. Lignification of the middle lamella in *L. decidua* revealed a heat-acclimative potential that likely contributed to reduced cuticular water loss. In *P. abies*, despite an obvious increase in cuticle thickness, no significant acclimative change was seen in *g*_min_. Flavonoids (kaempferol) were detected throughout the cuticle and outer cell wall of both species, but their contribution to reduced *g*_min_ is still unresolved. Our results demonstrate that cuticle and cell wall changes in conifer seedlings are species-specific and trait-dependent, and they highlight the role of epidermal chemistry in shaping resilience to climate warming at the alpine treeline.

## Introduction

At the alpine treeline, tree seedlings face some of the most challenging environmental conditions for growth and survival. In contrast to mature trees, seedlings grow close to the soil surface, and their needles can reach temperature maxima of >40 °C (Tiloca et al. 2025*a*), which is close to the heat survival limit (44–50 °C Larcher 2003). Across the alpine life zone, the heat risk for low-statured plants was shown to be highest at the treeline (Ladinig et al. 2015). Species with prostrate shoots may heat up to 20–40 K above air temperature (Salisbury and Spomer 1964, Körner and Cochrane 1983, Gauslaa 1984, Nagy et al. 2003, Larcher et al. 2010, Neuner and Buchner 2012, Ladinig et al. 2015). Resilience against heat is therefore a critical trait influencing whether tree seedlings can establish and persist at this ecological boundary.

Heat often coincides with drought. During heatwaves, plants typically close their stomata entirely to minimize water loss. Nevertheless, water can still escape through the cuticle and incompletely closed stomata. These pathways together define the leaf minimum conductance (*g*_min_; Schuster et al. 2017, Duursma et al. 2019). Increasing evidence suggests that *g*_min_ is a key factor influencing plant water use under heatwave conditions (Kala et al. 2016). A recent study showed that evergreen seedlings of treeline conifers, despite experiencing higher heat loads (+14 K) and higher baseline cuticle permeability than adult needles, still better resisted extreme heat-induced water loss due to a lower *g*_min_ (Tiloca et al. 2025*a*). Their cuticle traits varied by species and additionally revealed greater plasticity to thermal extremes than mature trees. Strikingly, seedlings lacked a distinct outer cuticle layer but had internal aromatic layers comparable to the needles of adults (Tiloca et al. 2025*a*). While deciduous needles of *Larix decidua* seedlings had the thinnest cuticles and more aromatic compounds, the evergreen needles of *Picea abies* and *Pinus cembra* seedlings had thicker and lipid-rich cuticles, which could also be relevant for survival of winter conditions.

In the alpine life zone, fine-scale microtopography, in addition to plant stature, can cause substantial variation in plant temperatures (Scherrer and Körner 2010, Neuner and Buchner 2012), with microsite exposure significantly affecting cuticle structure and function (Tiloca et al. 2025*b*). Indeed, in the study on needles of conifers of Tiloca et al. (2025*a*) seedlings were investigated at a site with north-western exposure. The observed differences between the needles of mature trees and those of seedlings growing under different thermal conditions may reflect the acclimative plasticity of the species, but ontogenetic effects are also likely to play a role. To resolve whether heat stress drives acclimative changes in the cuticle architecture and properties, we exposed treeline conifer seedlings to a controlled in situ heat treatment. We tested the following hypotheses: (i) long-term exposure of conifer tree seedlings to heat induces an increase in cuticle thickness; (ii) significant structural and microchemical modifications occur and (iii) these changes lead to reduced water loss through the cuticle (lower *g*_min_) in heat-acclimated seedlings compared with controls when exposed to heat. We also expected (iv) these heat-acclimative responses of the cuticle to be species-specific, given that the study species—deciduous *L. decidua* and evergreen *P. abies*—differ greatly in cuticle thickness, structure and chemistry. Understanding the physiological mechanisms involved in the survival of tree seedlings in heat may provide key insights into their survival potential at the alpine treeline, which is important for the future distribution of tree species in a changing climate.

## Materials and methods

### Plant material and experimental site

Seeds (cold stratified for at least 8 weeks) of the deciduous *L. decidua* Mill. and the evergreen *P. abies* (L.) H. Karst were obtained from the Tyrolean State Forest Garden in Nikolsdorf, Tyrol, Austria. At the experimental site (Zell am See, Austria, 47°18′24″N/12°47′29″, 785 m a.s.l.)—a horizontally orientated and largely open site that receives sunlight from early morning through the afternoon—200 seeds per species were used. The seeds were sown in early summer (on 26 June 2023) in small Polyvinyl chloride (PVC) pots (7 × 7 × 8 cm) filled with alpine soil to which mycorrhizal fungi (Ektomyk TREE, Terrafit GmbH, Pfaffendorf, Germany) had been added. Germination began relatively uniformly after 10–13 days (germination rate approx. 80–90%). After emergence, the seedlings were watered regularly. Once they had reached a height of 1–2 cm, half of the seedling boxes were kept under natural field conditions at the study site, while the other half were exposed to a controlled heat treatment. Seedlings from the heated and unheated plots were selected at random. The seedlings were ~6–8 cm tall at the time of harvest. They were cut at a height of 5 cm above the ground, and the whole bunch of seedling needles was used to measure minimum diffusive conductance (*n* = 27 per species and treatment). For confocal Raman measurements, the entire seed boxes were transferred to the laboratory in Vienna (Austria) at the end of the heat treatment, and the seedlings were placed inside plastic vials at −20 °C. For each species, three independent seedlings per treatment (control and heated) were used, resulting in a total of six seedlings per species.

### Controlled *in situ* heat treatment of conifer seedlings

To study the impact of long-term heat exposure during seedling development on *g*_min_ and the leaf cuticle structure, seedlings of *P. abies* and *L. decidua* were subjected to elevated temperatures for 6 weeks (31 July to 12 September 2023). The seedlings, which had recently germinated and were around 1–2 cm tall, were placed in a cold frame greenhouse made of transparent Plexiglas (Juwel Easy Fix, Juwel, Imst, Austria). The cold frame greenhouse was automatically heated by eight Positive Temperature Coefficient (PTC) heaters (14 × 18×4 cm, 12 V/130 W; PTC 21, Renkforce, Conrad, Hirschau, Germany) and cooled via 120 mm diameter openings in the side walls. These openings were fitted with 12 V/1800 rpm fans (FAN103, LogiLink, Schalksmühle, Germany), which were switched on when cooling was required. Three thermocouple sensors (GG-TI-28, Omega Engineering Inc., Stamford, CT, USA) were regularly distributed inside the cold-frame greenhouse at seedling height (5 cm) to measure the actual ambient air temperature. A data logger combined with a multiplexer (CR1000 and AM25T, Campbell Scientific, Logan, UT, USA) controlled the mean air temperature by automatically switching the power supply to the heaters and fans on and off (control interval: 2 s), to keep the average air temperature 10−15 K above the immediate ambient air temperature, which was measured by an NTC-sensor, which was placed in the shade outside of the cold frame greenhouse (107 temperature probe, Campbell Scientific). Unheated controls were exposed to the natural conditions at the experimental study site in close distance (<1 m) to the cold frame greenhouse.

At the same time, six fine-wire thermocouple sensors (wire diameter 0.08 mm; TT-TI-40, Omega Engineering Inc.) per species were mounted to the abaxial side of the primary leaves of the investigated species with super glue (UHU Blitzschnell Supergel, Bolton Adhesives, Milan, Italy). Heating was only applied during the day (ambient Photosynthetic Photon Flux Density (PPFD) >100 μmol photons m^−2^ s^−1^; SKP 215, Campbell Scientific). To prevent heat damage to the leaves, the heating (set 10–15 K above ambient air) was automatically deactivated for 1 min as soon as one of the 12 measured leaf temperatures exceeded 42 °C. This is still 2 K lower than the lowest heat killing temperature reported for conifer needles (Schulze et al. 2002).

### Determination of the minimum diffusive conductance

The actual diffusive conductance (*g*) was determined on 13 Sepember 2023 by measuring the mass loss of the seedlings over time (see Pisek and Berger 1938, Cape and Percy 1996). For this purpose, the well-watered seedlings, bearing tender cotyledons (typically 5–12) as well as primary leaves (typically 15–30), were cut off at the hypocotyl, which was then immediately sealed with candle wax (90 °C) to prevent water loss through the cut hypocotyl. The weight of these samples (*n* = 27 per species) was then repeatedly (typically in 30–60 min intervals) determined (BA 210S, Sartorius, Göttingen, Germany), while atmospheric parameters, such as the ambient air temperature (*T*) and relative humidity (Rh) (DK-RF400, Drießen & Kern, Bramstedt, Germany) and current atmospheric air pressure (*p*) (GFS 3000, Walz, Effeltrich, Germany) were measured in parallel. Assuming that the relative humidity in the intercellular spaces of the mesophyll was almost 100%, the *g* was then calculated directly from (i) the atmospheric parameters and (ii) from the mass loss between two consecutive weighings, which corresponds to the average transpiration rate (von Willert et al. 1995). To enable rapid work, a template was created for a spreadsheet programme (MS Excel, Microsoft, Redmond, WA, USA) in which only the atmospheric parameters and the measured mass had to be entered. After the mass measurements had been completed, leaf area was determined precisely from the scanned leaves (ImageJ 1.53 t, National Institutes of Health, USA). The template automatically calculated the molar vapour pressure deficit (VPD) between the interior of the leaf and the ambient air from the atmospheric parameters (*T*, Rh, *p*), and the transpiration rate (*E*) from the mass loss between two consecutive weighings. Analogous to Ohm’s law, the diffusion conductivity *g* was then calculated by dividing VPD by *E*. As the Excel template immediately displayed the actual diffusion conductivity for each individual sample in graphical form, the temporal course of *g* could be tracked in real time. Usually, *g* initially decreases rapidly because of immediate stomatal closure but finally (typically after 60–120 min) reaches a minimum value that does not change for several hours. This value was calculated as the minimum diffusion conductance (*g*_min_) (for a detailed description, see Tiloca et al. 2025*b*).

In order to determine a possible temperature-dependent response of *g*_min_, during the whole bench drying procedure samples of both species were exposed to either 25 °C or 41 °C. Bench drying was conducted within the cooling compartment of a switched-off laboratory freezer. Its lid was replaced by a transparent plexiglass-plate, and the internal temperature was kept constant at either 25 °C or 41 °C using PTC-heaters (12 V/100 W, Tru Components C-MZ45-FJ120-12 V-ZK, Conrad Electronics, Wels, Austria). The heaters were controlled by a special control unit (HTTS; Heat Tolerance Testing System; Buchner et al. 2013).

### Confocal Raman microscopy and spectral analysis

Before measurements started, seedlings were removed from the −20 °C freezer. By use of fine tweezers, three independent seedlings were sampled. From each seedling, one needle was collected (three needles per treatment) and cut in half. The dissected needles were mounted on a polyethylene glycol 600 (PEG, Carl Roth, Germany) filled sample holder. Cross sections (16–18 μm thick), cut with a cryo-microtome (CM 3050 S, Leica Biosystems, Germany), were transferred to glass slides and rinsed three times with distilled water to remove PEG. Sections were covered with a 22 × 22 mm coverslip (170 ± 5 μm, Marienfeld, Germany) and sealed with quick-drying nail polish to prevent dehydration and movement during measurements. Raman measurements were carried out with a confocal Raman microscope (alpha300, WITec Oxford Instruments, Germany) equipped with a piezo scanner, a 532 nm polarized laser (WITec Oxford Instruments), a spectrometer (UHTS 300 VIS) and a CCD detector. Spectra were recorded through a 100× oil-immersion objective (NA = 1.4, Zeiss Microscopy). To minimize chlorophyll fluorescence a bleaching scan of a large area was performed (2 μm step size, 0.00001 s integration time, 30 mW). The subsequent measurement was included inside the bleached area, at intervals of 0.3 μm, with an integration time of 0.05 s and laser power set to 20 mW. Data acquisition and spectral processing were conducted using WITec Plus software (version 6.0; WITec Oxford Instruments). Pre-processing was conducted in WITec Plus 6.0, including wavenumber selection (400–3100 cm^−1^), cosmic ray removal (filter size: 2, dynamic factor: 4.5) and baseline correction (ninth-order polynomial, zero noise threshold). Raman images were generated using the package True Component Analysis (TCA, WITec Oxford Instruments). This package uses an algorithm, that distinguishes chemically distinct regions and identifies spectral components at every pixel to generate a spatial map of the hyperspectral dataset. A residual image guided the extraction of the components until there were no significant spectral differences. For each region, an average spectrum was calculated from the most representative pixels, where those represent a chemically distinct region, though they might share components from neighbouring regions (e.g., mixed components). The identified regions were merged in a singular image (combined Raman imaging), from which spectra, corresponding to the same colour-coded areas, were extracted and averaged, to show an average spectrum.

A detailed spectral analysis was performed by extracting spectra from the specific regions within the measurement maps using band integration. For each species and treatment, 14 measurements were analysed (seven from the control and seven from the heated, distributed across the sampled needles) by the analysis tools in WITec Plus 6.0 (WITec Oxford Instruments). To enhance the view of the cuticle and epidermal cell region, a filter was applied targeting the CH region centred at 2900 cm^−1^ with a band width of 160 cm^−1^. Using a pen selection tool, silhouettes of the targeted areas were manually traced and then iteratively expanded by dilation until the full desired region was included. Four distinct anatomical regions were identified: two outermost layers (continuous cuticle layer—including the outermost layer in *L. decidua*—and outer epidermal cell wall) and two inner layers (middle lamella with primary cell wall, and secondary cell wall) (see Figure S1 available as Supplementary Data at *Tree Physiology* Online). Because the Raman spectra of the middle lamella and primary wall were nearly identical (both showing overlapping signals of lignin and polysaccharides), we merged them into a single region. The lignin band originated mainly from the interface between the middle lamella and the primary walls of adjacent cells, while the secondary cell wall was spectrally distinct, dominated by cellulose signals. The merged region thus represents the combined primary wall-middle lamella–primary wall complex. Spectra from these regions were extracted and imported into Unscrambler ×10.3 (CAMO Software AS., Oslo, Norway) to analyse spectral differences using principal component analysis (PCA). The dataset was organized both by individual anatomical region and by species to evaluate potential interspecific differences. Two-dimensional score plots of the first two principal components (PC1 and PC2) were generated, as these components capture the largest proportion of variance in the data. Corresponded loading plots were used to identify spectral features that contributed most strongly to the observed separation among samples. A Hotelling’s *T*^2^ ellipse (95% confidence limit) was overlaid on the score plots to aid in detecting potential outliers. When outliers were detected, the analysis was carried out again until no outliers remained.

### Statistical analysis

For each species and treatment, 12 Raman images were acquired (six from the control and six from the heated, with three images per needle surface). These images were obtained from the sampled needles, with three images per needle surface analysed. The images were processed into ImageJ/Fiji (Schindelin et al. 2012) to quantify the thickness of the most continuous cuticle layer. At least nine random thickness measurements per image were taken. The resulting dataset was processed in R (RStudio Posit Team 2023) to address three effects: (i) differences in adaxial (upper, US) and abaxial (lower, LS) needle side, across species and treatment (control vs heated), (ii) differences in cuticle thickness of the two conifer species (*L. decidua, P. abies*) and (iii) treatment effects (control vs heated) within species. Cuticle thickness was analysed as a continuous response variable. To account for repeated measurements, we applied generalized linear mixed-effects models (GLMMs) with a Gaussian error distribution using the glmmTMB package (Brooks et al. 2017). Each needle originated from a different seedling; therefore, needle identity represents the level of biological replication. Needle identity was included as a random intercept to account for non-independence of measurements within individual plants. The baseline intercept was set by the alphabetically first species (*L. decidua*). For differences of needle side, models included needle side, species and treatment as fixed effects. For differences in cuticle thickness, we focused on species differences, applying a likelihood ratio test that evaluated species prediction on cuticle thickness. Treatment effects were tested within species, again including side as a covariate. Model performance and assumptions were evaluated by visual inspection of residuals with the *DHARMa* package (Hartig 2024). Post hoc pairwise contrasts of estimated marginal means were calculated using the *emmeans* package (Lenth 2025). Results are presented as effect sizes in micrometres (μm), with 95% confidence intervals and associated *P*-values.

To assess differences in *g*_min_ between heated and unheated individuals of both species, the individual *g*_min_ data (each *n* = 27) were checked for normal distribution by the Kolmogorow–Smirnow test followed by pairwise comparisons (Mann–Whitney U-test, *P* < 0.01) using IBM SPSS Statistics 27, Armonk, NY, USA.

## Results

### Temperatures during the controlled long-term heating

**Figure 1:**
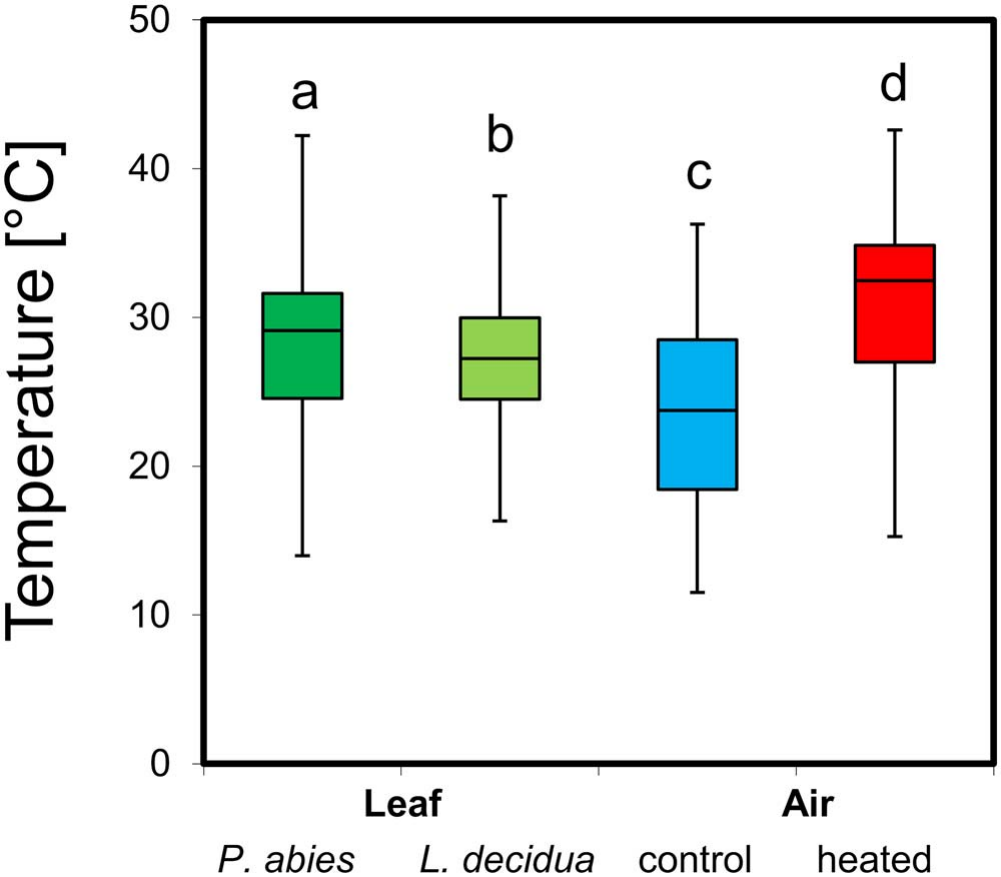
Daytime needle temperatures (°C) of tree seedlings of *P. abies* (green) and *L. decidua* (light green) that were exposed to a controlled in situ heat treatment inside of a cold frame greenhouse from 31 July to 12 September 2023. In order to generate a strong high temperature stimulus without damaging the needles, air temperature inside the greenhouse (red) was programmed to be ~10−15 K higher than the ambient air outside (blue) with a set maximum for individual needle temperatures of 42 °C. Boxes show the temperatures during daylight hours (PPFD >100 μMol photons m^−*2*^s^−1^; 25th, 50th and 75th percentiles, whiskers extend to maximum of 1.5 ICR). Different lower-case letters indicate significant differences in the mean values (*P* < 0.001, Kruskal–Wallis test) (needle temperatures per species *n* = 17,298, air temperatures per approach *n* = 2883).

During daytime long-term heating, average needle temperatures (±SD) of *P. abies* and *L. decidua* seedlings were 28.3 ± 5.0 °C and 27.1 ± 4.3 °C, respectively (Figure 1). Meanwhile, the ambient air temperature inside the heated cold frame greenhouse was 31.2 ± 5.4 °C, while outside in the control plot it was 23.6 ± 5.9 °C. These differences were all statistically significant (*P* < 0.001). Maximum needle temperatures of 42.9 °C (*P. abies*) and 41.5 °C (*L. decidua*), alongside maximum air temperature of 42.6 °C inside the cold frame greenhouse and of 36.3 °C outside (close to the unheated control group) were measured.

### Effect of heat treatment on g_min_

**Figure 2:**
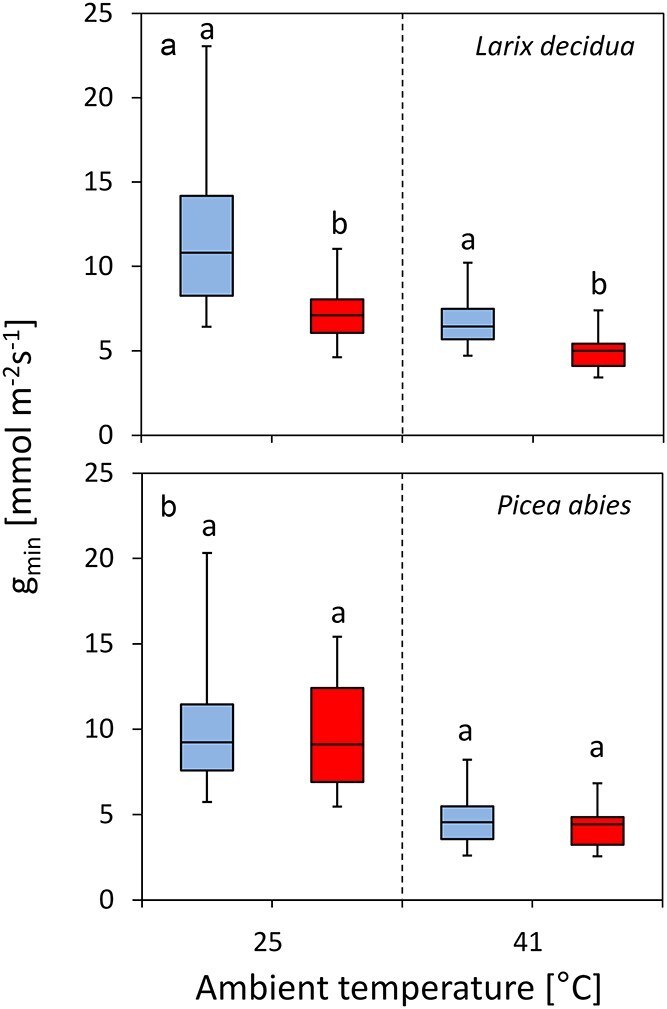
Minimum needle diffusive conductance (*g*_min_) measured at either 25 °C or 41 °C on needles of tree seedlings of (a) *L. decidua* and (b) *P. abies* that had either been cultivated for 6 weeks (31 July to 12 September 2023) under natural conditions (blue boxes) or under controlled heat exposure (set to 10–15 K above the control ambient air) inside a cold frame greenhouse (red boxes). The heating was automatically switched off as soon as a single needle temperature reached 42 °C. Statistically significant differences between *g*_min_ of the heated seedlings and the unheated control group are indicated individually by different lower-case letters (Mann–Whitney U-test; *n* = 27, *P* < 0.01). Boxes show the 25th, 50th and 75th percentiles. Whiskers extend to maximum 1.5 of Interquartile Range (IQR).

For both species, the exposure temperature (25 °C or 41 °C) during bench drying significantly affected the measured *g*_min_ values. Whether the samples had undergone long-term heating prior to the measurement or came from the unheated control group was irrelevant (Figure 2; see Table S1 available as Supplementary Data at *Tree Physiology* Online). However, the two species responded differently to long-term heating: whilst *g*_min_ of *L. decidua* was significantly lower (*P* < 0.01) in the long-term heated leaves than in the unheated ones (Figure 2a), regardless of whether the exposure temperature during the determination of *g*_min_ was 25 °C or 41 °C, *g*_min_ of *P. abies* needles did not differ significantly (*P* > 0.05) at any temperature (Figure 2b).

### Cuticle and cell wall microchemistry

**Figure 3:**
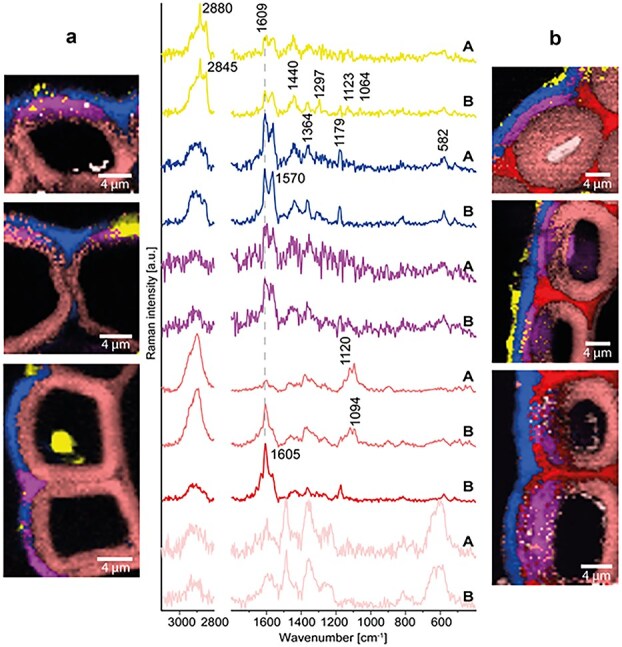
Combined Raman imaging of *L. decidua* control (a) and heated (b) seedlings showing cuticle and epidermal layers. Spectra from the outermost cuticle layer reveal a mixture of long-chain alkanes and aromatic components (yellow) in both treatments. Beneath, layers enriched in flavonoid-associated bands (blue, magenta), are more defined in heated samples. Heated samples exhibited thickened epidermal cell walls (pink, red, b) with Raman spectra showing both cellulose and lignin, whereas control seedlings displayed thinner cell walls dominated by cellulose. Lignin in the middle lamella was only detected in heated seedlings and suggested localized lignification as part of acclimation response. Other internal components were found in both treatments but resulted unidentified (light pink).

**Figure 4:**
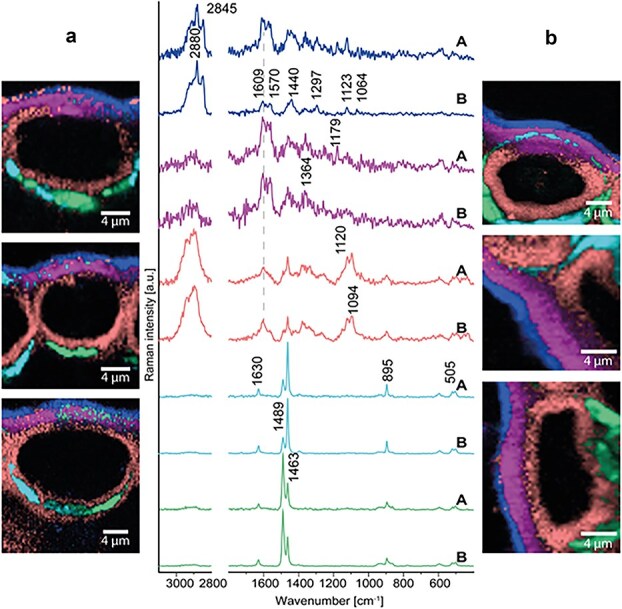
Combined Raman imaging of *P. abies* control (a) and heated (b) seedlings showing cuticle and epidermal layers. The outermost cuticle layer shows in the spectra a mixture of long-chain alkanes and aromatic components (blue) in both treatments. The subjacent layer is enriched in flavonoids (magenta), and more pronounced in heated samples. Heated samples displayed thicker epidermal cell walls (pink, b) characterized by Raman bands indicative of cellulose. In addition, calcium oxalate monohydrate (cyan, aquamarine), was detected in the primary and secondary cell wall and, most prominently, along the middle lamella of heated samples.

Combined Raman images from both species and treatments, processed with TCA, revealed a cuticle composed of one to two layers (yellow and blue, Figures 3 and 4) connected to the outer epidermal cell wall layer (magenta, Figures 3 and 4) and further to the epidermal cell wall (pink and red, Figures 3 and 4). In both species the cuticle consisted of a continuous aromatic-lipidic layer (Figures 3a, b and 4a, b, blue), with *L. decidua* additionally exhibiting an outer alkane layer (Figure 3a and b, yellow). The epidermal cell wall appeared thicker in the heated samples of both species (Figures 3a, b and 4a, b, pink) and showed the most pronounced differences in chemical composition: lignified middle lamella was detected exclusively in heated *L. decidua* samples (Figure 3b, red), while calcium oxalate crystals occurred in both control and heated *P. abies* samples (Figure 4a and b, cyan and aquamarine)*.*

In *L. decidua*, the outermost cuticle layer was irregularly present in both control and heated samples, as indicated by sharp CH-region peaks at 2880 and 2845 cm^−1^, along with characteristic long-chain alkane bands at 1297, 1123 and 1064 cm^−1^ (Czamara et al. 2015) (Figure 3a and b, yellow). The same bands were found in *P. abies* cuticles, but as part of continuous cuticle layer spectra (Figure 4a and b, blue). In both species, this layer alternated between aromatic and lipidic spectral contribution. The peak at 1609 cm^−1^ indicated aromatic compounds in both species (Varsányi 1969), while additional bands at 1570, 1364, 1179 and 582 cm^−1^ distinguished spectral signature of kaempferol (Sasani et al. 2021). A strong band at 1440 cm^−1^, indicative of cutin (Colthup and Daly 1990), was present in both cuticle layers but absent from the outer epidermal cell wall. The high intensity of the 1609 and 1570 cm^−1^ bands confirmed the presence of flavonols in this layer (Krysa et al. 2022) (Figures 3a, b and 4a, b, magenta). Furthermore, the cell wall layer displayed cellulose bands at 1094 and 1120 cm^−1^ (Wiley and Atalla 1987), in all species and treatments. This region appeared thicker in the heated samples, particularly in the middle lamella of *L. decidua*, where the TCA algorithm identified a component characterized by a high intensity 1605 cm^−1^ band, attributed to lignin (Figure 3b, red) (Bock and Gierlinger 2019). In the primary and secondary cell walls of both *P. abies* treatments, bands at 1489, 1463, 895 and 505 cm^−1^ identified calcium oxalate in its monohydrated form (Murata and Kawai 1956, Edwards et al. 1997). The 1489 and 1463 cm^−1^ bands showed variations in intensity, and the TCA algorithm extracted what appeared to be two different components (Figure 4a and b, cyan and aquamarine), but these were attributed to variations in crystal orientation rather than chemical differences. Even without chemical differences, minor changes in orientation of the crystal can alter the relative intensities of specific vibrational modes due to polarization selection rules (Tuschel 2020). These deposits occasionally extended into the middle lamella and outer epidermal cell wall layers.

### Spectral and chemical variability by PCA

**Figure 5:**
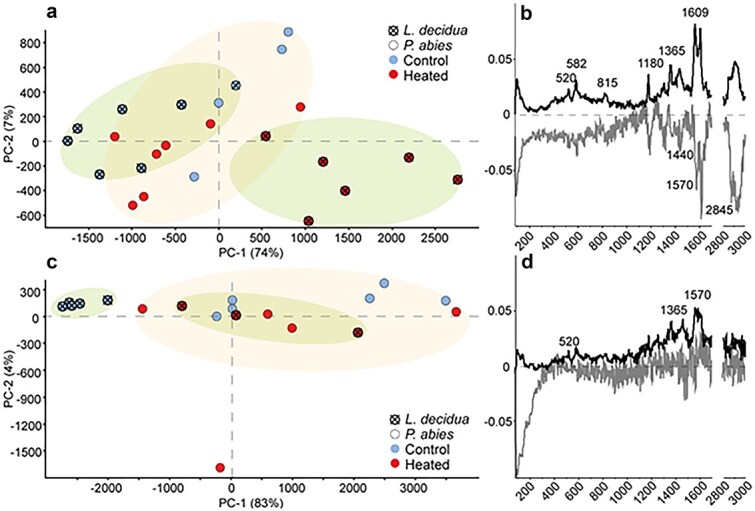
PCA of Raman spectra from cuticle and outer epidermal cell wall regions of *L. decidua* and *P. abies* seedlings under control and heated treatments. (a) PCA score plot of cuticle spectra showing separation along PC1 (74%) between *L. decidua* control (blue crossed circles) and heated (red crossed circles) samples, while *P. abies* control (blue circles) and heated (red circles) largely overlap. (b) PC1 loadings highlight bands assigned to aromatic compounds, suggesting that flavonoids contribute to the observed separation in *L. decidua*. PC2 (7%) explained less variance and contained mixed lipid and aromatic signals. (c) PCA score plot of outer epidermal cell wall spectra with *L. decidua* again separating control and heated samples along PC1 (83%). (d) PC1 loadings indicate aromatic contributions, whereas PC2 (4%) was noisy and could not be clearly assigned. Defined cuticle regions are shown in Figure S1, available as Supplementary Data at *Tree Physiology* Online.

**Figure 6:**
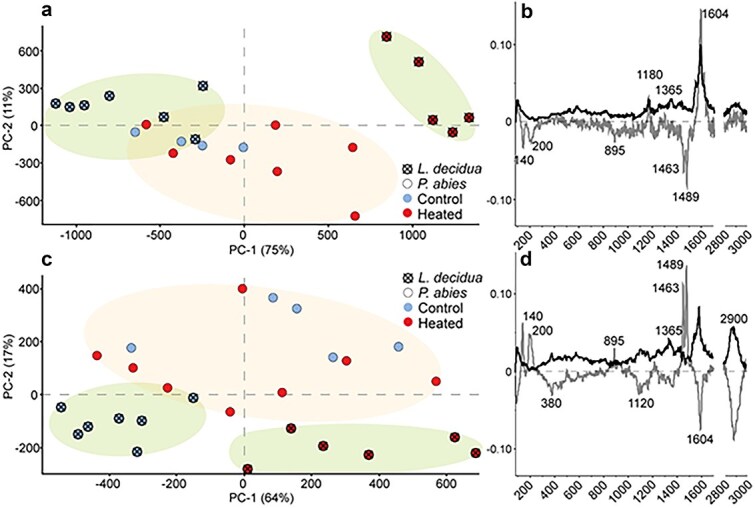
PCA of Raman spectra from epidermal cell wall regions of *L. decidua* and *P. abies* seedlings under control and heated treatments. PCA score plot of primary wall plus middle lamella (A) and secondary cell wall (C) spectra shows separation along PC1 (A: 75%, C: 64%) between *L. decidua* control (blue crossed circles) and heated (red crossed circles) samples, while *P. abies* control (blue circles) and heated (red circles) largely overlap. (B) PC1 loadings highlight positive lignin bands, suggesting that lignin contributes to the observed separation in *L. decidua*. PC2 (A: 11%, C: 17%) explained less variance but highlighted the presence of calcium oxalate (B) and cellulose (D) in the spectra. Defined epidermal regions are shown in Figure S1, available as Supplementary Data at *Tree Physiology* Online.

The PCA analysis of extracted Raman spectra from *L. decidua* and *P. abies* revealed distinct species-specific compositional responses to heat treatments across the cuticle, epidermal cell wall and deeper cell wall regions (Figures 5 and 6). In *L. decidua*, heated and control samples formed clusters along PC1 in all examined regions (Figures 5a, b and 6a, b), while *P. abies* spectra resulted in overlapping clusters with no consistent separation, and indicated no consistent chemical response to the heat treatment. In the cuticle and outer epidermal cell wall, *L. decidua* loadings for PC1 were characterized by positive peaks at 1609, 1365, 1180, 815, 582 and 520 cm^−1^ consistent with aromatic compounds (Figure 5b). Heated samples consistently plotted on the positive side along PC1 and suggested an enrichment of these compounds in the selected regions. PC2 explained only minor portion of the variance (7%) and was dominated by noisy signals, but included bands tentatively assigned to lipids and aromatics (e.g., 2845, 1570 and 1440 cm^−1^). In the middle lamella plus primary wall (Figure 6b), and secondary wall region (Figure 6d), PC1 loadings displayed dominant lignin-associated bands (e.g., 1604, 1365 and 1180 cm^−1^), with heated *L. decidua* again clustered on the positive side along the axis. PC2 loadings of these regions (11% and 17% variance explained) contained bands at 1489, 1463, 895, 200 and 140 cm^−1^ (Figure 6b−d), characteristic of calcium oxalate monohydrate. These bands were associated with *P. abies*, which, despite exhibiting these signals, still showed overlap in the score plots (Figure 6a and b) and thus a non-heating response. PC2 loadings also had bands at 2900, 1604, 1120 and 380 cm^−1^ indicative of a mixture from lignin and cellulose.

### Cuticle thickness

Cuticle thickness did not vary significantly between the adaxial and abaxial needle sides, with only a difference of 0.01 μm (*P* = 0.90, Table 1; see Table S2 available as Supplementary Data at *Tree Physiology* Online). Species identity had a strong effect on cuticle thickness (χ^2^ = 48.0, *P* < 0.001, see Table S2 available as Supplementary Data at *Tree Physiology* Online). When considering control and heated treatments together, *L. decidua* exhibited cuticles that were on average 0.58 μm thicker than those of *P. abies* (see Table S2 available as Supplementary Data at *Tree Physiology* Online). In the control seedlings, cuticle thickness was slightly higher in *L. decidua* (1.67 μm) than in *P. abies* (1.23 μm), with a difference of 0.44 μm. Heat exposure caused a marked thickening in *L. decidua* needles (from 1.67 to 2.39 μm, +0.72 μm, *P* < 0.001, Table 1; see Table S2 available as Supplementary Data at *Tree Physiology* Online), and a smaller but significant increase in *P. abies* (from 1.23 to 1.67 μm, +0.44 μm, *P* < 0.001, Table 1; see Table S2 available as Supplementary Data at *Tree Physiology* Online).

**Table 1:** Statistical differences in cuticle thickness across needle sides (adaxial, US and abaxial, LS), species (*L. decidua* and *P. abies*) and treatments (control vs heated). The statistical summary shows estimated values, standard error (SE), degrees of freedom (DF), 95% confidence interval bounds (CI lower, CI upper) and *P*-values. Mean cuticle thickness values (μm) are given for each species.

			Estimated	SE	DF	CI lower	CI upper	*P*
**Needle side**	LS−US		0.01	0.08	207	−0.15	0.17	0.90
**Species**	*Larix−Picea*		0.58	0.08	206	0.41	0.74	<0.001
**Treatment**	*L. decidua*							
	Control (μm)	Heated (μm)	−0.72	0.14	204	−1.00	−0.45	<0.001
	1.67	2.39						
	*P. abies*							
	Control (μm)	Heated (μm)	−0.44	0.10	204	−0.64	−0.23	<0.001
	1.23	1.67						

## Discussion

Our study addressed key hypotheses regarding the effects of long-term heat exposure on conifer seedlings driven by species-specific cuticle and cell wall changes. Both *L. decidua* and *P. abies* developed thicker cuticles under heat, but only *L. decidua* exhibited a reduction in *g*_min_, which was underpinned by flavonoids in the cuticle and lignification of the middle lamella. In contrast, *P. abies* displayed calcium oxalate crystal deposition in both treatments, but no significant differences in cuticular conductance.

During the long-term heat treatment, either *L. decidua* or *P. abies* maintained average needle temperatures lower than the surrounding air inside the cold frame greenhouse, which is a clear indication for transpirational cooling. Maximum needle temperatures recorded in the seedlings (~40 °C) remained well below the reported lethal thresholds for conifer needles (LT_50_ values of 44.4, 47.7 °C for *L. decidua* and *P. abies*, respectively, Neuner and Buchner 2023), and throughout the heat treatment, the seedlings continued to grow, producing new needles. This shows that the experienced temperatures were clearly sublethal and stressed the plants without causing irreversible heat damage.

In our study, heat exposure did not substantially alter the water barrier function of waxes in either species, and the presence or absence of epicuticular waxes alone could not explain the observed differences in *g*_min_. *Larix decidua* exhibited characteristic bands of long-chain alkanes in both treatments and as a separated cuticle layer, whereas in *P. abies* these compounds were incorporated into the most continuous cuticle layer. Under high temperatures or in climates with high vapour pressure deficits, leaves are often observed to accumulate epicuticular waxes (Bueno et al. 2022). A recent study on alpine populations of *Arabidopsis arenosa*, where at high elevation plants grow under higher evaporative demand, showed reduced cuticular transpiration in conjunction with a consistently altered cuticular wax composition, with higher accumulation of two fatty alcohols and two iso-alkanes (Bertel et al. 2025). While this increased deposition had an effect on *g*_min_, several studies show that it does not necessarily translate into lower cuticular permeability (Riederer and Schreiber 2001, Jetter and Riederer 2016, Bueno et al. 2020), even when their deposition increases manyfold (Grünhofer et al. 2022). A potential explanation comes from Garen and Michaletz (2025), who argued that at high temperatures, thermal expansion and phase transition in the cuticle cause to open additional water-transport pathways. In addition to heat-changes in epicuticular waxes happening at 35–70 °C (Schreiber and Schönherr 1990, Jetter 2006), this can thereby reduce the effectiveness of these compounds as a barrier to water diffusion. Thus, in *P. abies,* the embedding of alkanes into the cuticle matrix may enhance thermal resistance and maintain a tight water barrier, consistent with its stable and low *g*_min_ values. In contrast, the more exposed epicuticular waxes of *L. decidua* may undergo partial reorganization under heat, but the potential instability of these compounds may be compensated by more internal chemical reinforcements and thicker cuticles.

Raman imaging revealed a continuous cuticle layer characterized by typical bands of aromatics and lipids in both species and treatments. The PCA analysis further indicated that heated samples of *L. decidua* were positively associated with flavonoids. Their accumulation within the cuticle and epidermal layer can theoretically alter surface hydrophobicity and, through interactions with the cutin matrix, contribute to water movements towards the atmosphere. However, current evidence for a direct role of flavonoids in reducing *g*_min_ in this species is inconclusive and appears to depend more on the physicochemical compatibility between flavonoid nature, cuticular matrix and interaction with environmental factors (Tiloca et al. 2025*a*, 2025*b*). Particularly in this direction, heat stress has been shown to elevate total flavonoid content, as observed in leaves of sweet basil (Al-Huqail et al. 2020) and in whole tomato seedlings (Alhaithloul et al. 2021). Flavonoids enhance the photoprotective capacity of plant tissues and act as antioxidants that mitigate damage from reactive oxygen species generated from thermal stress. The spectral bands attributed to flavonoids in our samples are consistent with kaempferol (Sasani et al. 2021), a compound known to increase under enhanced UV-B radiation (Olsson et al. 1998). If we consider the experimental location and the increase of UV radiation with elevation (Körner 1999, Schmucki and Philipona 2002), along with the observed reduction in *g*_min_ when comparing mature vs seedlings conifers (Tiloca et al. 2025*a*), the presence of flavonoids in the cuticle and outer epidermal cell wall can plausibly be seen as a photoprotective defence mechanism against strong irradiation. However, when implemented within the cutin matrix, cuticular waxes and flavonoids act as fillers (Petracek and Bukovac 1995, Takahashi et al. 2012, Khanal et al. 2013, Tsubaki et al. 2013, España et al. 2014) potentially making very thin cuticles (<3 μm) more effective in water retention. This aligns with both control and heated *P. abies* cuticles, which show no differences in their *g*_min_. Nevertheless, their direct contribution of flavonoids to reduced *g*_min_ remains unresolved, likely depending on structural compatibility with cutin and waxes, and interaction with abiotic and/or biotic factors (Tiloca et al. 2025*a*, 2025*b*). Future research should focus on elucidating compatibility between flavonoids and other cuticle components, and their interactions with environmental factors that might mediate their influence on water permeability.

In both species subjected to heat treatments, we observed a consistent thickening of epidermal cells, with Raman spectra showing both cellulose and lignin bands in the primary and secondary cell wall but lignin alone in the middle lamella of *L. decidua*. Lignin is a phenolic compound deposited during secondary wall thickening that provides structural support by cross-linkages with cellulose and hemicellulose to reinforce the whole plant body (Chabannes et al. 2001). Because cellulose is hydrophilic, whereas lignin is hydrophobic, their balance affects water interactions and permeability with the cell wall. In conifers, lignification of the outer epidermal cell wall promotes water clusters and retention of water content and permeability but reduces dehydration as it retains water when drying (Reina et al. 2001). The thickened walls of heated samples therefore suggest an acclimative response to elevated temperature that enhance rigidity and cuticle-cell wall continuity. Any potential influence of lignin on *g*_min_ would likely be indirect, mediated through its contribution to mechanical integrity rather than by direct changes in water diffusion pathways. In an evolutionary context, plants established the transpiration barrier along with more mechanistically stable components (Zeisler and Schreiber 2016, Zeisler-Diehl et al. 2018). As a deciduous species with a short growing season, *L. decidua* may rely on the lignified middle lamella to retain water and withstand heat stress. In contrast, the evergreen *P. abies* did not show clear lignification sites in the epidermis but instead exhibited accumulation of calcium oxalate crystals. These crystals extended through the middle lamella and along the secondary wall, but on both heated and control seedlings. Their accumulation sites are consistent with earlier reports (Fink 1991), with abundance and hydration state varying with needle age (Fink 1991). However, we cannot confirm an increase of crystal accumulation for the heat treatments, nor an effect on *g*_min_ as the species showed no significant differences between the treatments. It is possible that, because cuticular conductance in *P. abies* is already intrinsically low, the species may have reached a physiological threshold below which further reduction in *g*_min_ is not attainable. In this context, our results raise the question of whether elevated temperatures accelerate the functional ageing of *L. decidua* needles, whereas *P. abies* maintains its baseline state despite increased cuticle thickness without clear acclimative shifts seen in *g*_min_.

Long-term heat exposure induced a significant increase in cuticle thickness in both *L. decidua* and *P. abies*. While our original expectation, based on prior work (Tiloca et al. 2025*a*), was for *L. decidua* to develop thinner cuticles than *P. abies*, the current study showed that heated seedlings of both species developed significantly thicker cuticles. This had repercussion on cuticular water permeability, with only the deciduous species exhibiting significant differences between the heated and control seedlings. Despite longstanding evidence that cuticle thickness alone does not influence *g*_min_ (Schreiber and Riederer 1996, Riederer and Schreiber 2001, Schuster et al. 2017, Bueno et al. 2020), our results indicate that cuticle thickness contributes significantly when integrated with chemical and epidermal cell wall changes, both of which improve water retention under prolonged warming.

Taken together, our findings reveal contrasting acclimative strategies in deciduous and evergreen conifer seedlings. *Larix decidua* responded to long-term heating with thicker cuticles and lignification, all of which contributed to a lower *g*_min_ under heat. *Picea abies*, on the other hand, exhibited cuticle thickening but no clear acclimative change in *g*_min_, reflecting its inherently low permeability. These differences mirror broader life-history strategies: deciduous larch favours plastic, short-term adjustments to cope with summer stress, while evergreen spruce maintains stable, conservative cuticle properties across seasons. Species response is highly trait-dependent, with contrasting strategies (e.g., deciduous vs evergreen) and epidermal chemistry shaping resilience (Henn et al. 2024). Placed in a more ecological perspective, these acclimative changes occur against a backdrop of climate-driven treeline dynamics. Warming generally promotes seed germination and early growth but often impedes seedling establishment because of high evaporative demand and reduced soil moisture (Marcante et al. 2014, Frei et al. 2018, Lett and Dorrepaal 2018, Palosse et al. 2024). Our findings support the hypothesis that *g*_min_ is capable of adjusting to drier environments and rising temperatures (Wang et al. 2025). This perspective underscores the importance of integrating cuticular physiology into models of seedling establishment under future warming scenarios, particularly at sensitive ecological boundaries like the alpine treeline.

## Conclusion

Long-term heat exposure induced thicker cuticles in both *L. decidua* and *P. abies*, but only heated *L. decidua* showed reduced cuticular water permeability, which was supported by lignification of the middle lamella. These findings highlight contrasting acclimation strategies between deciduous and evergreen conifers, with the deciduous larch exhibiting plastic adjustments to cope with heat, and evergreen spruce maintaining a conservative, low-permeability cuticle. Placed in an ecological context, our results suggest that species-specific responses are likely to be critical for seedling survival at the alpine treeline under future warming.

## Supplementary Material

Supplementary_material_tpag080

## Data Availability

The data and materials are available upon request.
