# Supplementary material for: Effect of long-term heat stress on structure and function of epidermal tissues in needles of treeline conifer seedlings
Source: Tree Physiol. 2026 Jun 11;46(7):tpag080. doi: 10.1093/treephys/tpag080 (PMC13358877; doi:10.1093/treephys/tpag080)
Supplement: Supplementary_material_tpag080 [file supplementary_material_tpag080.zip › Suppl Figure 1 heated seedlings.docx]

***
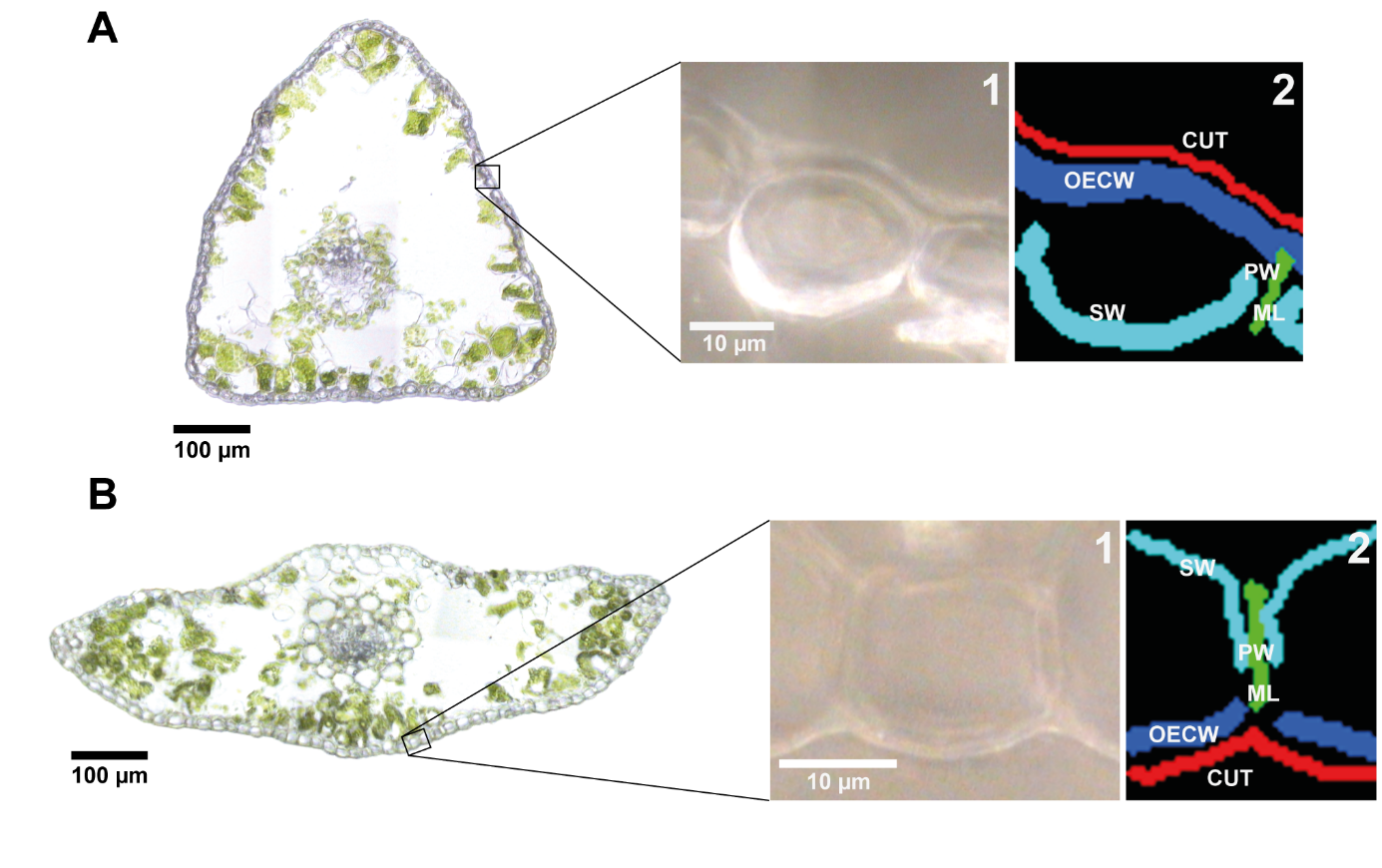
***

**Suppl. Fig. 1** Light microscopy cross-sections of needles from Picea abies (A) and Larix decidua (B) used to define regions of interest for Raman spectral extraction by band integration. Insets show higher magnification images (1) alongside schematic overlays (2) and identify specific tissue regions: cuticle (CUT, red), outer epidermal cell wall (OECW, dark blue), primary wall (PW, green), secondary wall (SW, cyan), and middle lamella (ML, green). These assignments were used to extract Raman spectra and analysed with Principal Component Analysis.
