# Supplementary material for: Effect of long-term heat stress on structure and function of epidermal tissues in needles of treeline conifer seedlings
Source: Tree Physiol. 2026 Jun 11;46(7):tpag080. doi: 10.1093/treephys/tpag080 (PMC13358877; doi:10.1093/treephys/tpag080)
Supplement: Supplementary_material_tpag080 [file supplementary_material_tpag080.zip › Suppl_Tables_12_heated_seedlings_11052026.docx]

Supp. Table 1. Minimum diffusive conductance (g_min_, mmol m⁻² s⁻¹) of Picea abies and Larix decidua seedlings measured at two exposure temperatures (25 °C and 41 °C) under control and long-term heated conditions. Values represent means ± SD. Statistical comparisons were performed between exposure temperatures within treatments, and between control and heated groups at each temperature. Significant differences are indicated as P values.

|  | Control | | | Heated | | | Sign. (heated vs. control) | |
| --- | --- | --- | --- | --- | --- | --- | --- | --- |
| Exposure temperature (°C) | 25 | 41 | Sign. | 25 | 41 | Sign. | 25 | 41 |
| g_min_ (mmol m⁻² s⁻¹)  *P. abies* | 9.8 ± 2.9 | 4.8 ± 1.5 | P<0.001 | 10.4 ± 5.9 | 4.3 ± 1.2 | P<0.001 | P>0.05 | P>0.05 |
| g_min_ (mmol m⁻² s⁻¹)  *L. decidua* | 11.9 ± 4.8 | 6.8 ± 1.5 | P<0.001 | 7.5 ± 2.2 | 5.4 ± 2.1 | P<0.001 | P<0.001 | P<0.001 |

Supp. Table 2. Statistical results from the generalized linear mixed model (glmmTMB). Cuticle thickness from control and heated Larix decidua and Picea abies is tested including both abaxial and adaxial needle sides. A likelihood ratio test (LRT) was applied to compare the model with and without species identity, giving AIC, BIC, X2 and p as a summary.

|  |  | **Estimate** | **SE** | **Z-value** | **P** |
| --- | --- | --- | --- | --- | --- |
| **Needle side** | Intercept | 2.32 | 0.14 | 15.98 | <2e^-16^ |
|  | Side | -0.01 | 0.08 | -0.12 | 0.90 |
|  | Species | -0.58 | 0.08 | -7.00 | 2.46e^-12^ |
|  | Treatments | -0.58 | 0.08 | -7.01 | 2.43e^-12^ |
| **Species** |  | **AIC** | **BIC** | **Χ^2^** | **P** |
|  | No-species | 454.67 | 474.86 |  |  |
|  | species | 410.67 | 437.60 | 47.99 | 3.78e^-11^ |
| **Treatment** |  | **Estimate** | **SE** | **Z-value** | **P** |
|  | Intercept | 2.39 | 0.13 | 18.23 | <2e^-16^ |
|  | Species | -0.72 | 0.15 | -4.91 | 9.15e^-07^ |
|  | Treatment | -0.72 | 0.14 | -5.23 | 1.72e^-07^ |
|  | Side | 0.004 | 0.07 | 0.05 | 0.96 |
|  | Species x treatment | 0.29 | 0.17 | 1.67 | 0.09 |
